# DIFFERENTIAL ASSOCIATIONS OF HAND GRIP STRENGTH AND CHAIR STAND TIME WITH INDOOR AND OUTDOOR FALLS AMONG OLDER ADULTS

**DOI:** 10.1093/geroni/igad104.1953

**Published:** 2023-12-21

**Authors:** Wenjun Li

**Affiliations:** University of Massachusetts Lowell, Lowell, Massachusetts, United States

## Abstract

Hand grip strength and chair stand time are physical performance measures that are predictive of frailty and muscle strengths in older age. We examined their associations with indoor and outdoor falls among older adults. The Healthy Aging and Neighborhood Study enrolled 397 community-dwelling older adults aged 65-95 years old in Central Massachusetts (2018-2020). Hand grip strength (in kilograms) and chair stand time (in seconds) were measured at baseline by trained research staff. Falls were reported on monthly falls calendar, and when a fall was reported, the circumstances were collected via telephone interview. Negative binomial regression models were used to estimate the associations of hand grip strength and chair stand time with both indoor and outdoor falls, separately. Models were adjusting for sex, age, BMI, bodily pain, comorbidities, ADL and IADL as appropriate. Overall, annual rates (95% CI) of indoor and outdoor falls were 40.2 (33.7-47.6) and 45.3 (38.4-53.1) per 100 person-years respectively. Stronger hand grip strength was associated with lower rate of indoor falls (IRR (95% CI): 0.94 (0.91-0.98) per kilogram). Longer chair stand time was associated with higher rate of indoor falls (IRR (95% CI): 1.06 (1.03-1.09) per second). There was no significant association of outdoor falls with grip strength (p=0.504) or chair stand time (p=0.430). In conclusion, hand grip strength and chair stand time were predictive of indoor falls but not outdoor falls. Future studies should examine the underlying mechanisms of such differential associations indoor vs. outdoor falls.

